# Graph Attention Feature Fusion Network for ALS Point Cloud Classification

**DOI:** 10.3390/s21186193

**Published:** 2021-09-15

**Authors:** Jie Yang, Xinchang Zhang, Yun Huang

**Affiliations:** 1 School of Geography and Planning, Sun Yat-sen University, Guangzhou 510275, China; yangj257@mail2.sysu.edu.cn; 2 School of Geography and Remote Sensing, Guangzhou University, Guangzhou 510006, China; 3 School of Software, Jishou University, Zhangjiajie 427000, China; huangyun@jsu.edu.cn

**Keywords:** ALS point cloud, classification, deep learning, graph attention mechanism, receptive field

## Abstract

Classification is a fundamental task for airborne laser scanning (ALS) point cloud processing and applications. This task is challenging due to outdoor scenes with high complexity and point clouds with irregular distribution. Many existing methods based on deep learning techniques have drawbacks, such as complex pre/post-processing steps, an expensive sampling cost, and a limited receptive field size. In this paper, we propose a graph attention feature fusion network (GAFFNet) that can achieve a satisfactory classification performance by capturing wider contextual information of the ALS point cloud. Based on the graph attention mechanism, we first design a neighborhood feature fusion unit and an extended neighborhood feature fusion block, which effectively increases the receptive field for each point. On this basis, we further design a neural network based on encoder–decoder architecture to obtain the semantic features of point clouds at different levels, allowing us to achieve a more accurate classification. We evaluate the performance of our method on a publicly available ALS point cloud dataset provided by the International Society for Photogrammetry and Remote Sensing (ISPRS). The experimental results show that our method can effectively distinguish nine types of ground objects. We achieve more satisfactory results on different evaluation metrics when compared with the results obtained via other approaches.

## 1. Introduction

Airborne laser scanning (ALS), also known as airborne light detection and ranging (LiDAR), is an important active remote sensing technique that has displayed rapid development in recent years [1]. The technique has the advantage of quickly acquiring large-scale, high-density, and high-precision 3D ground data, and plays an increasingly important role in many applications, including topographic mapping [2], urban planning [3], forest biomass estimation [4], environmental monitoring [5], and power line detection [6]. By employing ALS for ground-based scanning, a massive and disordered point cloud can be obtained. Classifying LiDAR point clouds is a basic and key step in the application of LiDAR data. Classifying ALS point clouds with high precision and high efficiency remains a challenging task due to the irregular distribution of point clouds and the complexity of scenes [7,8].

Point cloud classification usually involves assigning a category label to each point, which is often referred to as point cloud semantic segmentation in computer vision. In early studies, researchers classified point clouds by employing hand-engineered features and traditional classifiers [9,10,11,12] or preprocessed the point clouds before classification [13,14]. These methods belong to traditional machine learning methods, which fail to learn high-level features, whereas the methods based on deep learning techniques can further improve the classification accuracy due to the ability to learn high-level features. Many works [15,16] transform 3D point clouds into 2D images or 3D grids and then use deep learning techniques for classification; however, the transformation leads to information loss and a high computation cost. To avoid these problems, some studies directly process raw points while employing deep learning techniques, such as PointNet++ [17], SPG [18], and RandLA-Net [19]. The latter two networks have achieved good results in the large-scale point cloud classification task, which is considerably challenging. Many works capture more local features of the point cloud data by introducing a graph neural network [20] and a graph attention mechanism [21]. For example, GACNN [22] achieved a better performance than that of some prevalent networks on ALS point cloud datasets.

Due to the fact that encoder–decoder architecture can capture hierarchical semantic information, it has been successful in many 2D image semantic segmentation tasks. Many prevalent networks [17,21] also employ it for 3D point cloud semantic segmentation. The architecture needs appropriate methods for downsampling and feature extraction. Farthest point sampling (FPS) is widely used as a downsampling method, but it has a high time complexity of O(*N*^2^). This implies that the method is not suitable for point cloud datasets with a large number of points [19]. The graph neural networks for point cloud classification can efficiently capture the local structure information of point clouds, but the receptive field size of many graph neural networks is usually not sufficient to capture comprehensive contextual information.

Based on the above analysis, we present GAFFNet, a graph neural network that adopts encoder–decoder architecture. We abandon the expensive downsampling methods, such as FPS, and adopt the voxel grid downsampling, which balances efficiency and performance in order to help the feature extraction module to obtain features at different levels. On the basis of simplifying the preprocessing steps, we design a new feature extraction module. For ALS point cloud classification, our network achieves good results with a high efficiency. Our main contributions are as follows:

(1) We present GAFFM, a new feature extraction module based on the graph attention mechanism. The module increases the receptive field for each point and fuses the features of different scales. Therefore, the module can effectively capture wider contextual features at different levels;

(2) We propose GAFFNet, and the experimental results verify its effectiveness for ALS point cloud classification. We achieve more satisfactory results on the ISPRS semantic 3D dataset when compared with the results of other methods;

(3) GAFFNet has strong adaptability. It reduces the preprocessing steps and improves the efficiency. Due to the fact that our network is insensitive to the number of point clouds, this advantage allows us to directly feed the point cloud blocks with different numbers of points to the network after dividing the training set into point cloud blocks, and it also allows the trained network to be directly applied to test sets with different numbers of points without additional preprocessing.

The remaining part of this paper is organized as follows: in Section 2, a brief summary of the research related to our work is given; Section 3 presents GAFFNet in detail; experiments are performed in Section 4, and we evaluate the performance of GAFFNet and other methods; finally, we provide the conclusion in Section 5.

## 2. Related Work

Early studies on ALS point cloud classification mainly relied on hand-engineered features, using the unsupervised, supervised, or combination methods. The unsupervised method sets certain rules and divides the ground objects into a few categories [23,24]. This kind of method is highly dependent on the threshold, and therefore has poor adaptability. The supervised method provides hand-engineered features to traditional machine learning algorithms [9,10,11,12] for classification. This method does not have the ability to learn high-level features; in fact, it is difficult to further improve the classification accuracy. The combination method usually preprocesses the point clouds in unsupervised ways [13,14] and then uses supervised traditional machine learning algorithms to classify the point clouds. This kind of algorithm improves the classification accuracy to a certain extent; however, its processing flow is complex.

Deep learning has gradually become one of the most important technologies in pattern recognition, computer vision, and other fields in recent years [25], and point cloud classification methods based on deep learning have gradually emerged. According to the different input data formats for a neural network, the point cloud classification methods based on deep learning can be divided into three types: multiview-based, voxel-based, and point-based methods. The first two methods [15,16,26] transform 3D point clouds into 2D images or 3D grids and then use 2D CNN or 3D CNN to process them. However, information loss is inevitable, and the voxel-based method is not suitable for large-scale point clouds because of its high computation cost. The point-based method directly processes the raw points. As a pioneer work, PointNet [27] uses MLP and max pooling to extract global features of point clouds, but it is difficult to fully capture the contextual information. Then, PointNet++ [17] was developed, which represents an improvement on PointNet, as it employs a hierarchical neural network; it achieved good results. Inspired by PointNet/PointNet++, some researchers have proposed other networks [28,29].

In recent years, researchers have used a graph structure to represent point clouds, and are attempting to employ the graph neural network to classify point clouds [20,30]. Wang et al. [31] proposed DGCNN using a graph structure to capture local geometric information while ensuring permutation invariance. More recently, the attention mechanism has been paid more attention, and various research has introduced it in order to learn a more adaptive local relationship of point clouds. Inspired by GAT [32], GACNet [21] captures the relationship information between points through the graph attention mechanism, thereby allowing one to achieve better classification results. GACNN [22] performs better than other prevalent networks (e.g., PointNet++ and GACNet) on ALS point cloud datasets through its graph attention convolution module, which can learn local structural features and global contextual information. However, many graph neural networks have a problem where the receptive field size is not sufficient to capture comprehensive contextual information.

**Figure 1:**
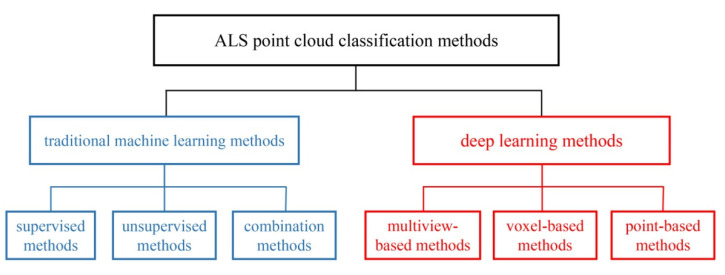
A common classification of ALS point cloud classification methods.

Figure 1 shows the different ALS point cloud classification methods intuitively. The significant difference between traditional machine learning methods and deep learning methods is that the former generally relies on low-level features, such as hand-engineered features, whereas the latter has the ability to learn high-level features to improve the classification accuracy. In addition, PointNet and its improved methods, as well as the methods based on the graph neural network, belong to point-based methods because they directly process the raw points.

## 3. Methodology

### 3.1. Overview

Many ALS point clouds with more than one million points have a distribution range of hundreds of meters in length and width and diverse ground objects. The classification for these ALS point clouds is a huge challenge. We adopted the graph neural network based on encoder–decoder architecture to capture features of ALS point clouds at different scales. We employed the voxel grid downsampling method to obtain point clouds with different densities and then constructed a graph pyramid with those point clouds (Section 3.2). We also designed a graph attention feature fusion module (Section 3.3) based on the graph attention mechanism, which was used to capture wider semantic features of point clouds. Based on the above modules and methods, we designed a neural network (Section 3.4) that can effectively capture contextual features at different levels, which is required by the ALS point cloud classification task.

### 3.2. Graph Pyramid Construction

Graph pyramids with different scales were constructed by alternately preforming graph construction and graph coarsening on point clouds. The multi-scale graph pyramid can incorporate semantic information of point clouds at different scales, which helps to improve the network’s ability to classify point clouds. The specific steps of graph construction and coarsening are shown below.

#### 3.2.1. Graph Construction

For a given point cloud *P* = {*p_i_*|*i* =1, 2, …, *N*}, we first employed the *K*-nearest neighbors (KNN) algorithm implemented by KD-tree to search for the spatial neighbors of each point, and then we built a graph *G*(*V, E*), where *V* and *E* denote the nodes and the edges in the graph, respectively. The node *v_i_* ∈ *V* corresponds to the point *p_i_*. For the point *p_i_*, N*_i_* denotes its neighborhood set, and *p_ij_* represents a neighbor of point *p_i_*, where *j* ∈ N*_i_*.

#### 3.2.2. Graph Coarsening

We employed voxel grid downsampling [33] to implement graph coarsening and obtain a pyramid of downsampled point clouds. Specifically, for the input point cloud *P*, *P^l^* represents the subsampled point cloud, where *l* = {0, 1, …, *L*}, and *L* is the number of downsampling, *P*^0^ = *P*. We overlaid 3D voxel grids over the point cloud *P^l^* and then replaced all points inside the voxel grids with their centroid. Finally, we obtained the subsampled point cloud *P^l^*^+1^. For each *P^l^*, a corresponding graph *G^l^*(*V^l^, E^l^*) can be constructed, as described in Section 3.2.1.

### 3.3. Graph Attention Feature Fusion Module

Many previous works on point cloud classification based on the graph attention mechanism have achieved good results, but few of them have solved the problem that the receptive field size of the networks is limited. To this end, we propose a graph attention feature fusion module (GAFFM) to improve the performance of point cloud classification. It includes not only extracting the local contextual information from the neighboring points (Section 3.3.1), but also extracting the wider contextual information from the extended neighboring points (Section 3.3.2), which effectively increases the receptive field of the network and improves the performance of the network.

#### 3.3.1. Neighborhood Feature Fusion Unit

**Figure 2:**
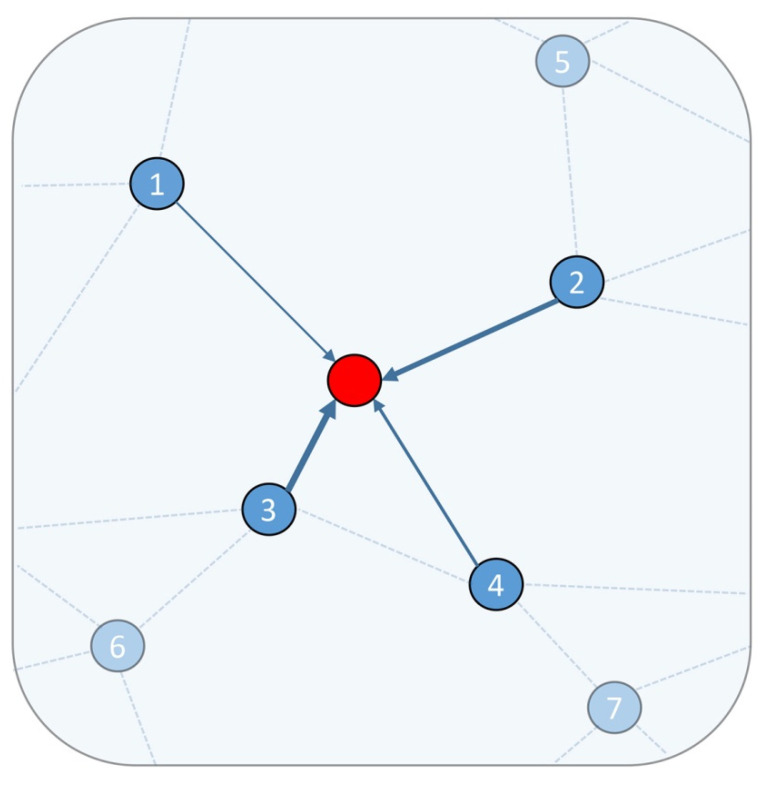
Illustration of the NFFU for a subgraph of a point cloud. The red point is a subsampling point, and is called the center point. The four points (point 1 to point 4) are the nearest neighbors of the central point. The features of the neighborhood set are aggregated to the center point by employing the NFFU. The blue solid line with different widths represents the different attention coefficients of each neighbor.

Inspired by [19,21], we designed a neighborhood feature fusion unit (NFFU) that can aggregate the information of neighboring nodes to the center node according to their attention weight. Figure 2 illustrates the NFFU for a subgraph of a point cloud. The unit first encodes the raw features and intermediate learned features of all neighboring nodes via MLP, and then it fuses the two encoded features to obtain the enhanced features. Then, we normalized the encoded enhanced features via the SoftMax function to obtain the attention coefficient and finally aggregated the features according to the attention coefficient. The statistical features can reflect the quantitative features of things as a whole, and the computation cost is low; therefore, we fused the statistical features of all neighboring nodes when encoding the raw features, which helped our NFFU to capture rich contextual information at different scales. The NFFU includes the following three steps.

(1) Searching for neighbors for all points and graph construction. Given a point cloud *P^l^*, we used the method in Section 3.2.2 to downsample the point cloud *P^l^* and obtain the subsampled point cloud *P^l^*^+1^, where *l* = {0, 1, …, *L*} and *L* is the number of downsampling. We call the point in the subsampled point clouds the center point. We used the KNN algorithm to identify the top *k* nearest neighbors in the point cloud *P^l^* for each center point in the point cloud *P^l^*^+1^, and then we constructed a KNN graph *G*(*V, E*). In addition, except for the geometric coordinates, other features of the center point are consistent with the nearest point identified in the point cloud *P^l^*;

(2) Calculation of the attention coefficient for all neighbors. This step includes four parts: encoding raw features, encoding intermediate learned features, fusing the above two features, and calculating the attention coefficient. The raw features of each center point and its neighbor are denoted as *r_i_* and *r_ij_*, respectively, and the feature vector includes x-y-z coordinates and intensity values, where *j*∈$\;N_{i}$ and $N_{i}$ is the neighborhood set of the center point. The intermediate learned features of each center point and their neighbors are denoted as *m_i_* and *m_ij_*, respectively. The encoded raw features, the encoded intermediate learned features, the enhanced features, and the attention coefficient of the neighbor are denoted as *R_ij_*, *M_ij_*, *E_ij_*, and *c_ij_*, respectively. They are calculated as follows:

*R*_*ij*_ = *MLP* (Δ*r*_*ij*_ ⊕ *d*_*ij*_ ⊕ Δ*S*_*ij*_) (1)

*M*_*ij*_ = *MLP* (*m*_*i*_ ⊕ Δ*m*_*ij*_) (2)

**Figure 3:**
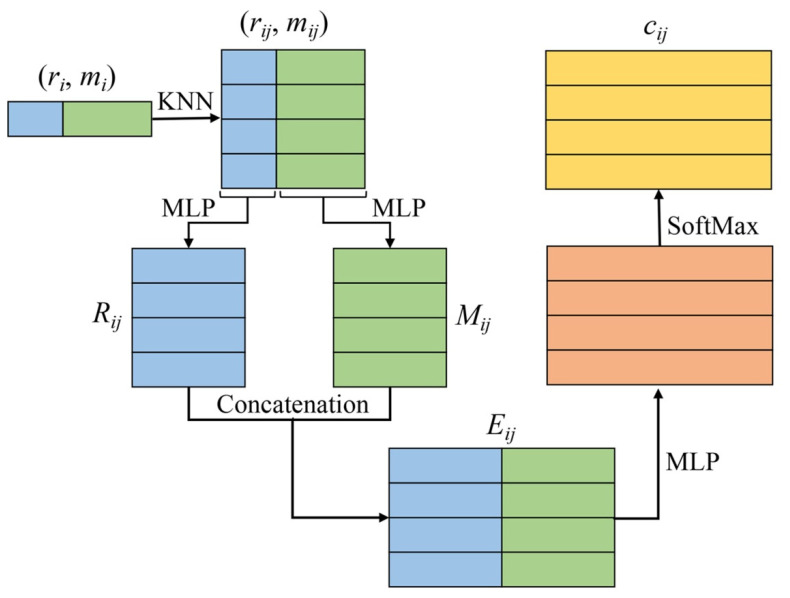
The mechanism employed in the NFFU for generating the attention coefficient of neighbors. The NFFU takes the raw features and intermediate learned features of neighbors as input, and then obtains the attention coefficient of neighbors through operations, including MLP, concatenation, and SoftMax.

*E*_*ij*_ = *R*_*ij*_ ⊕ *M*_*ij*_ (3)

(4) $$c_{ij}=\frac{\exp\;\left(\mathrm{LeakReLU}\left(MLP\;\left(E_{ij}\right)\right)\right)}{\sum_{j\in N_{i}}\;\exp\;\left(\mathrm{LeakReLU}\left(MLP\;\left(E_{ij}\right)\right)\right)}\;$$

where ⊕ is the concatenation operation, *MLP* represents the applied multi-layer perceptron, and *d_ij_* represents the Euclidean distance between the center point and its neighbor. Δ*m_ij_* = *m_ij_* − *m_i_*, Δ*r_ij_* = *r_ij_* − *r_i_*, Δ*S_ij_* = $\tilde{r}$*_ij_* − *S_i_*, *S_i_* represents the Z coordinate statistic values or intensity statistic values for the neighborhood set, and for efficiency, we only counted the maximum, minimum, median, and average. $\tilde{r}$*_ij_* is the Z coordinates or intensity of the neighbor. It appears that Equations (1) and (2) use redundant information, but this helps the network to learn richer information and obtain better performance. Figure 3 shows the mechanism employed in the NFFU for generating the attention coefficient of neighbors.

(3) Obtaining the attention feature for each center point. According to the attention coefficient of the neighbors, we weighted summed the enhanced features of the neighbors and obtained *H_i_*, the attention feature for each center point. The unit’s final output *H_i_* can be formulated as follows:

(5) $$H_{i}=\sum_{j\in N_{i}}\;(c_{ij}*E_{ij})\;$$

where ∗ represents the element-wise production.

#### 3.3.2. Extended Neighborhood Feature Fusion Block

For the point cloud *P^l^* and its subsampled point cloud *P^l^*^+1^, some important points in *P^l^* may be dropped after downsampling, which leads to the degradation of network performance. We need to increase the probability that the information of the points in *P^l^* will propagate to *P^l^*^+1^, allowing us to reduce the impact of some important points being discarded on the network performance. Inspired by [19,34], we simply stacked multiple NFFUs to form an extended neighborhood feature fusion block (ENFFB), which enabled us to reserve more information of points at a low cost.

**Figure 4:**
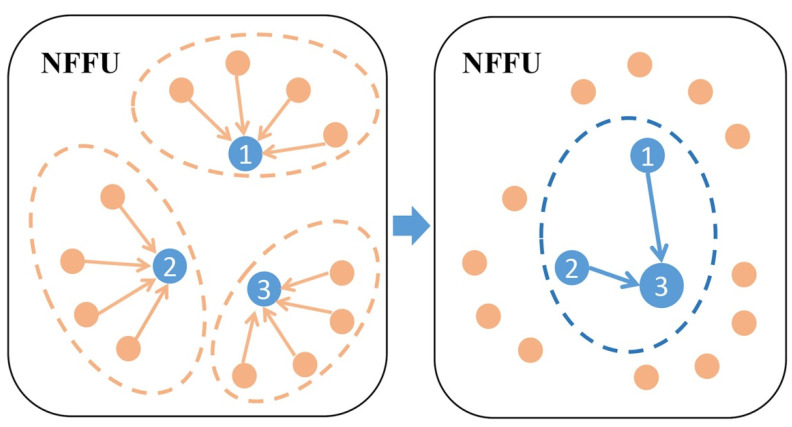
Illustration of ENFFB for a subgraph of a point cloud. The three points (point 1 to point 3) are the center points, and the orange points are their neighboring points. By applying NFFU twice, center point 3 not only aggregates the information of its neighboring points, center point 1 and center point 2, but also aggregates the information of the neighboring points of center point 1 and center point 2, which significantly increases the receptive field for center point 3.

As shown in Figure 4, our single NFFU takes the points in the subsampled point cloud *P^l^*^+1^ as the center points, and then searches for the neighboring points in the upper-layer point cloud *P^l^* to construct the KNN graph. Then, we constructed another KNN graph whose center points are the points in *P^l^*^+1^, and the neighboring points are also the points in *P^l^*^+1^. We employed NFFU again to propagate the information of points. By simply stacking multiple NFFUs, we significantly expanded the scope of information dissemination and increased the receptive field for each center point, which can improve the network performance.

**Figure 5:**
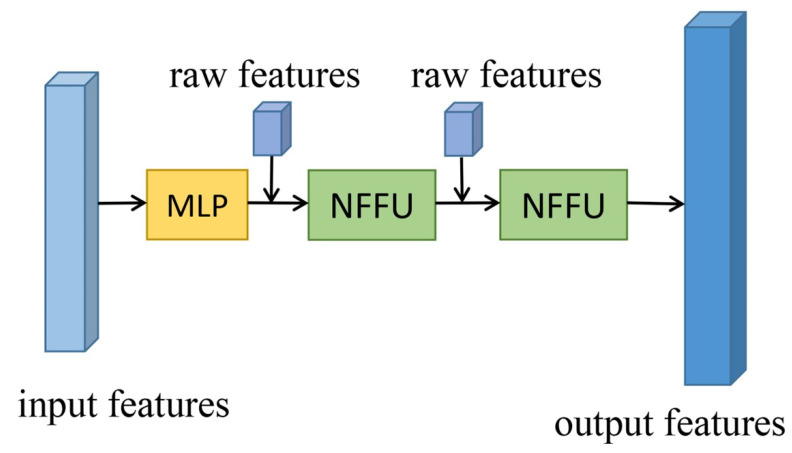
Architecture of the ENFFB, which includes MLP and multiple NFFUs. The raw features come from the ALS point cloud dataset and includes x-y-z coordinates and intensity values.

GAFFM can contain one or more ENFFBs, and a standard GAFFM contains one ENFFB and two NFFUs. Figure 5 illustrates the architecture of the ENFFB.

### 3.4. Graph Attention Feature Fusion Network

**Figure 6:**
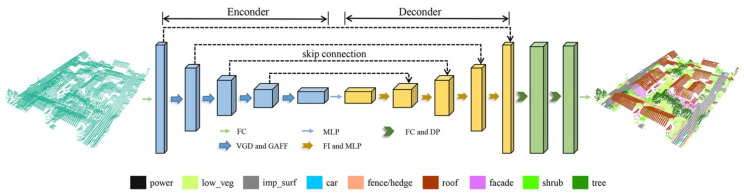
Architecture of GAFFNet: FC, fully connected layer; VGD, voxel grid downsampling; GAFF, graph attention feature fusion; MLP, multi-layer perceptron; FI, feature interpolation; DP, dropout.

We employed encoder–decoder architecture to construct the whole GAFFNet. It mainly includes the encoder layers, the decoder layers, and the classification layers. Figure 6 shows the detailed architecture of GAFFNet.

Our encoder network consists of one full connection (FC) layer and four encoder layers. Firstly, we fed the input point cloud to the FC layer. Then, we employed GAFFM to extract the features of points in each encoder layer after voxel grid downsampling and increased the feature dimension of each point. Finally, the encoder network captured the multiscale features of the point cloud.

Our decoder network consists of four decoder layers. To obtain the feature set that has the same number of points as that of the input point cloud, the inverse distance weighting method was employed for feature interpolation layer by layer in the decoder layers. More details of the interpolation method can be found in [17]. We concatenated the interpolated features with the features of points from the corresponding encoder layer through a skip connection. Then, we applied MLP to reduce the feature dimension of each point and finally obtained the features of points in each decoder layer.

The classification layer following the decoder layers was used to predict the final semantic label. The semantic label for each point was obtained through three FC layers and two dropout layers.

## 4. Experiments

### 4.1. Data Description

**Figure 7:**
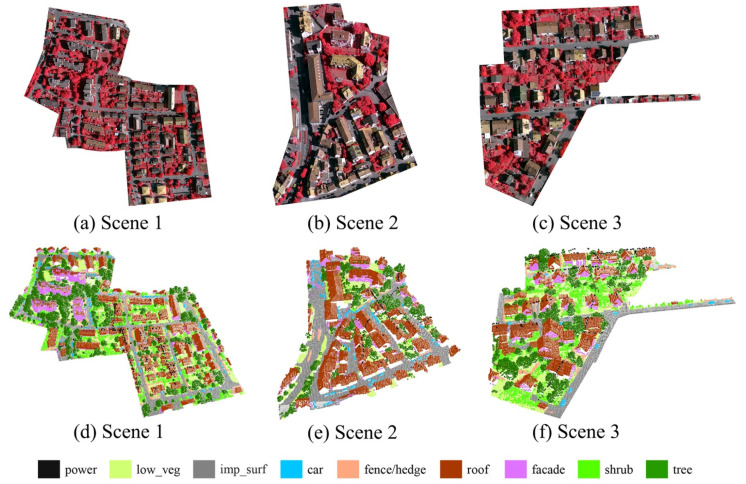
Three scenes of the experimental dataset. Scene 1 is used as the training set, and Scene 2 and Scene 3 are used as the test set. (**a**–**c**) are orthoimages; (**d**–**f**) are ALS point clouds.

We evaluated the performance of our network on the ALS point cloud dataset provided by the International Society for Photogrammetry and Remote Sensing (ISPRS). The dataset was obtained in August 2008 by a Leica ALS50 system [35]. ISPRS provides this dataset from Vaihingen (Germany) as a benchmark dataset for 3D semantic labeling. The ISPRS 3D dataset, which consists of 1,165,598 points, contains nine categories [12], namely, powerline (power), low vegetation (low_veg), impervious surfaces (imp_surf), car, fence/hedge, facade, roof, shrub, and tree. As shown in Figure 7, the dataset covers three areas that correspond to three scenes. Scene 1 is used as the training set with 753,876 points, whereas Scene 2 and Scene 3 are used as the test set, and the test set has a total of 411,722 points. Table 1 and Table 2 show the number and the proportion of 3D points per category in the training set and the test set, and it can be seen that the distribution of categories in the dataset is extremely unbalanced, especially the powerline points, which only account for approximately 0.1%.

### 4.2. Implementation Details

Limited by GPU memory, it is almost impossible to directly feed the entire training set into the network. Therefore, we first divided the point cloud of the training set into the small point cloud blocks with the same size, and then fed them into the network. Due to the fact that our network is insensitive to the number of point clouds, the network allows the point cloud blocks with a different number of points to be directly fed, and also allows for the entire test set to be directly fed into the trained network. Specifically, the area of each point cloud block that is partitioned from the training set is 30 × 30 m, and each block overlaps its adjacent blocks; the distance between two adjacent blocks is 10 m in the *x*-axis or *y*-axis direction. The blocks whose number of points is less than the fixed number are not fed to the network. Furthermore, 10% of training blocks are selected as the validation dataset.

In addition to the features provided by the ISPRS 3D dataset, the input features for the network also include the height above the DTM feature, which is important for point cloud classification [12]. We employed the method proposed by [36] to obtain the height above the DTM feature for each point, and fed it into the network, where the DTM grid size was set to 0.8 m and the rigidness was set to 2. The input features for our network included x-y-z coordinates, intensity, the product of the number of returns and the return number, and the height above DTM.

Our network was implemented based on PyTorch, and we employed the Adam optimizer. The initial learning rate was set to 0.002 and decreased by 20% every ten epochs. All processing steps of our method (such as downsampling, KNN algorithm, etc.) are implemented by Python. The sizes of the voxel grid for downsampling were 0.6, 1.2, 2.4, and 4.8 m, and the K parameter in the KNN algorithm was set to 10. The hardware platform used in the experiment was equipped with Intel i9-9900K CPU, 32G memory (RAM) and a single NVIDIA RTX2080Ti GPU.

### 4.3. Experiment Results

**Figure 8:**
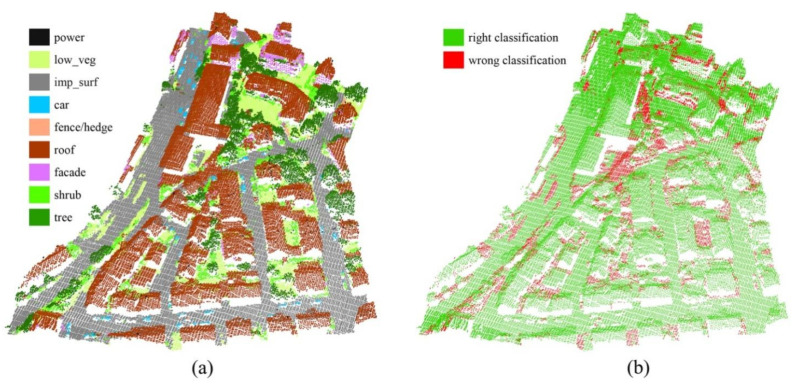
Classification results for Scene 2: (**a**) nine ground objects classified from Scene 2; (**b**) classification error map for Scene 2.

**Figure 9:**
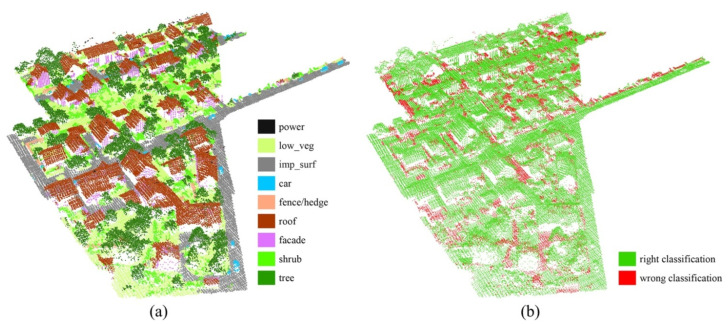
Classification results for Scene 3: (**a**) nine ground objects classified from Scene 3; (**b**) classification error map for Scene 3.

Figure 8 and Figure 9 show the classification results for Scenes 2 and 3 in the test set, respectively, and our method correctly labelled most of the points in the test set.

In addition, we quantitatively evaluated the classification results with the standard evaluation metrics used by the ISPRS 3D labeling contest. The results include the three metrics of accuracy, recall, and F1 score for each category and are shown in Table 3.

From Table 3, we can see that our method can correctly classify most of the test points. We achieved satisfactory classification results (F1 score higher than 75%) for five categories, namely, low vegetation, impervious surfaces, roof, car, and tree, most of which are large objects. For example, the precision, recall, and F1 score for the roof category are 93.9, 94.2, and 94.1%, respectively. Our method has poor classification results for the shrub and fence/hedge categories, which may be due to their similarity of low-level features, including height and geometric distribution features. In addition, some shrub points were wrongly labelled as trees and low vegetation, which may be due to the lack of clear boundaries between shrubs and the two categories.

### 4.4. Comparison with Other Methods

Due to the category imbalance for the ISPRS 3D dataset, it is not entirely reasonable to evaluate the network performance using only the overall accuracy (OA). We introduced the macro average F1 score (abbreviated as macro avg F1), which is the unweighted mean of the F1 scores for all categories. This metric assigns equal weight to each category and is insensitive to category imbalance. Therefore, it is more reasonable to combine the OA and macro avg F1 score to evaluate the classification performance for imbalance datasets.

We compared our method with six other existing methods published by the ISPRS organizers through the above evaluation metrics (see Table 4). The six methods, namely, UM [37], LUH [38], BIJ_W [8], RIT_1 [39], NANJ2 [40], and WhuY4 [41], have achieved the top six performances in terms of OA on the ISPRS 3D dataset. Among them, UM and LUH rely on hand-engineered features to classify point clouds, whereas the other four methods employ deep learning techniques for classification. Relatively speaking, the methods based on deep learning perform better than the other methods.

During the training step, the network can obtain a good OA performance by paying attention to the categories with a large number of points, because these categories have a considerable impact on OA performance, while ignoring the small categories that have little impact on OA performance. To obtain a good macro avg F1 score, we need to treat every category equally, including the small categories. However, too much focus on small categories tends to lead to overfitting and reduces OA performance. Therefore, it is not easy to balance the OA and macro avg F1 score.

**Figure 10:**
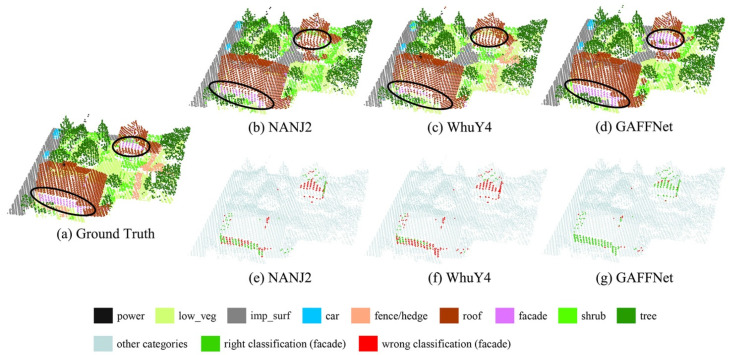
The classification results of the different methods and ground truth on a selected region: (**a**) ground truth; (**b**–**d**) nine ground objects classified by different methods; (**e**,**f**) classification results of different methods for the facade points. The black circles are the areas where the facade points gather.

As can be seen from Table 4, compared with other methods, our method well balances OA and the macro avg F1 score, and achieves the highest performance in terms of the macro avg F1 score. In addition, our method displays the best performance in terms of F1 scores for some categories. It is worth mentioning that our method achieves a significantly high classification performance for the facade category. To illustrate this intuitively, we plotted the classification results of three different methods in Figure 10, namely, NANJ2, WhuY4, and GAFFNet, which demonstrate the top three performances shown in Table 4. Facade points are easily covered and misclassified. As shown in Figure 10, the NANJ2 and WhuY4 methods misclassify many facade points into the roof category or other categories, and our method has a much larger number of correctly classified points (green) than that of the other two methods. This is most likely because the NANJ2 and WhuY4 methods lose the 3D spatial information when converting the point cloud into 2D images.

To evaluate the superiority of our method more comprehensively, we compared our method with more methods—specifically, the methods based on the graph neural network. We not only compared our method with GACNN [22], but also with the prevalent graph neural networks, such as GACNet [21], DGCNN [31], and GAT [32]. Those methods based on the graph neural network have all been proposed recently. The authors of [22] reported the classification results of GACNet and DGCNN for the ISPRS 3D dataset, and the results are shown in Table 5.

In a point cloud classification network, the feature extraction module is one of the most critical parts in determining the classification performance, but other parts (such as downsampling, graph construction, and preprocessing) can also affect the classification results. To demonstrate the superiority of our feature extraction module more fairly, we use improved versions of GACNet and DGCNN for comparisons. Specifically, for the improved versions, only the feature extraction modules were the same as the original networks, and other parts were the same as our method. The improved versions of GACNet and DGCNN are called GACNet-voxel and DGCNN-voxel, respectively. In addition, we also compared our method with GAT, which first proposed the graph attention mechanism. GAT was not applied to point cloud classification when it was proposed, so we dealt with it in the same way as for GACNet; the improved version is called GAT-voxel. The classification results of all the above methods are shown in Table 5.

Our method achieved a better OA performance than that of other methods and obtained a satisfactory macro avg F1 score (as shown in Table 5). In addition, our method achieved a better performance than that of GACNet (GACNet-voxel) and DGCNN (DGCNN-voxel) by a large margin. This implies the superiority of our feature extraction module. The reason for the lower performance of other methods may be due to the fact that their receptive field size is limited for sufficiently capturing semantic features.

It can be seen from Table 5 that the classification results of GACNet-voxel and DGCNN-voxel are not worse than those of their original versions. Furthermore, GACNN and the original GACNet employ the FPS for downsampling, which has a high time complexity, whereas our method and the improved versions all use voxel grid downsampling, which has a lower time complexity than that of the FPS.

### 4.5. Ablation Study

To evaluate the influence of various modules and features in GAFFNet, we conducted the following ablation experiments to demonstrate the effectiveness of GAFFNet:

(1~3) The addition of max/sum/mean pooling. After each GAFFM, we added the widely used max/sum/mean pooling;

(4) The replacement of voxel grid downsampling with random sampling. The downsampling method in our network was replaced by random sampling with a high time efficiency (abbreviated as GAFFNet-RS), and 25% points were retained after each random sampling;

(5~6) A change in the number of NFFU stacks. In GAFFNet, we stacked two NFFUs to form a standard GAFFM. In the ablation study, we conducted two groups of experiments with one NFFU or three NFFUs in a GAFFM;

(7) The removal of the height above the DTM feature. The computational cost is required to obtain the height above the DTM feature; therefore, we evaluated the impact of removing this feature on the network performance;

(8) The removal of statistical features. In our NFFU, we fused the statistical features, and in the ablation study, we evaluated the impact of removing this feature on the network performance.

Table 6 shows the OA and macro avg F1 scores for all ablation networks. From Table 6, we can make the following conclusions:

(1) The widely used max/sum/mean pooling tends to select features mechanically, so their performance is not necessarily optimal, and the mean pooling is the worst of the three;

(2) Random sampling can shorten the sampling time, but the distribution of sampling points is random; this method may lose part of the point cloud information, which leads to the performance loss of the OA and macro avg F1;

(3) The performance degrades regardless of whether there is one NFFU or three NFFUs in GAFFM. This may be due to the limited receptive field size of one NFFU, which makes it difficult to improve the performance, and the excessive number of trainable parameters for three NFFUs, which leads to overfitting;

(4) After removing the height above the DTM feature, the OA performance decreases, whereas the macro avg F1 score is the highest among all networks. Therefore, if we do not prioritize a high OA performance, we can remove this input feature;

(5) After removing the statistical features, the performance loss of the OA and macro avg F1 is considerable different: the former loss is small, and the latter loss is large.

## 5. Conclusions

For ALS point cloud classification, we propose GAFFNet, which is based on the graph attention mechanism, reducing the preprocessing steps. We use the graph attention network as the base network and design a new feature extraction module (i.e., GAFFM) that fuses multi-level features and effectively increases the receptive field size for each point with a low computational cost. Therefore, the module can effectively capture wider contextual features at different levels, which can improve the network classification performance. In addition, our GAFFNet is insensitive to the number of point clouds, which greatly reduces the corresponding preprocessing steps and improves the classification efficiency. In this paper, the superiority of our method is demonstrated via extensive experiments. To further improve the classification accuracy, we are planning to further mine the relationship between neighboring points in the neighbor set, which helps us to calculate the attention weight of each neighboring point more accurately.

## Figures and Tables

**Table 1:** The number of 3D points per category in the training set and the test set.

Dataset	Power	Low_Veg	Imp_Surf	Car	Fence/Hedge	Roof	Facade	Shrub	Tree
Training Set	546	180,850	193,723	4614	12,070	152,045	27,250	47,605	135,173
Test Set	600	98,690	101,986	3708	7422	109,048	11,224	24,818	54,226

**Table 2:** The proportion (%) of 3D points per category in the training set and the test set.

Dataset	Power	Low_Veg	Imp_Surf	Car	Fence/Hedge	Roof	Facade	Shrub	Tree
Training Set	0.07	23.99	25.70	0.61	1.60	20.17	3.61	6.31	17.93
Test Set	0.15	23.97	24.77	0.90	1.80	26.49	2.73	6.03	13.17

**Table 3:** Classification results of GAFFNet on the ISPRS 3D dataset.

Metrics	Power	Low_Veg	Imp_Surf	Car	Fence/Hedge	Roof	Facade	Shrub	Tree
Precision	0.768	0.850	0.894	0.883	0.678	0.939	0.632	0.441	0.770
Recall	0.475	0.789	0.940	0.691	0.234	0.942	0.578	0.454	0.879
F1	0.587	0.818	0.916	0.775	0.348	0.941	0.603	0.447	0.821

**Table 4:** Performance comparison between our method and six other methods on the ISPRS 3D dataset. Except for the numbers in the last two columns, which show the overall accuracy (OA) and macro avg F1 scores, the other numbers demonstrate the F1 scores of each category for different methods. The boldface text shows the best performance among the different methods.

Methods	Power	Low_Veg	Imp_Surf	Car	Fence/Hedge	Roof	Facade	Shrub	Tree	OA	Macro Avg F1
UM	0.461	0.790	0.891	0.477	0.052	0.920	0.527	0.409	0.779	0.808	0.590
LUH	0.596	0.775	0.911	0.731	0.340	0.942	0.563	0.466	**0.831**	0.816	0.684
BIJ_W	0.138	0.785	0.905	0.564	0.363	0.922	0.532	0.433	0.784	0.815	0.603
RIT_1	0.375	0.779	0.915	0.734	0.180	0.940	0.493	0.459	0.825	0.816	0.633
NANJ2	**0.620**	**0.888**	0.912	0.667	0.407	0.936	0.426	**0.559**	0.826	**0.852**	0.693
WhuY4	0.425	0.827	0.914	0.747	**0.537**	**0.943**	0.531	0.479	0.828	0.849	0.692
GAFFNet	0.587	0.818	**0.916**	**0.775**	0.348	0.941	**0.603**	0.447	0.821	0.841	**0.695**

**Table 5:** Performance comparison between our method and other methods based on the graph neural network on the ISPRS 3D dataset. Except for the numbers in the last two columns, which show the overall accuracy (OA) and macro avg F1 scores, the other numbers demonstrate the F1 scores of each category for different methods. The boldface text shows the best performance among the different methods.

Methods	Power	Low_Veg	Imp_Surf	Car	Fence/Hedge	Roof	Facade	Shrub	Tree	OA	Macro Avg F1
GAT-voxel	0.380	0.752	0.892	0.656	0.305	0.880	0.324	0.409	0.773	0.785	0.597
GACNN	**0.760**	0.818	**0.930**	**0.777**	**0.378**	0.931	0.589	0.467	0.789	0.832	**0.715**
GACNet	0.628	**0.819**	0.908	0.698	0.252	0.914	0.562	0.395	0.763	0.817	0.660
GACNet-voxel	0.444	0.794	0.903	0.704	0.355	0.918	0.480	**0.475**	0.812	0.820	0.654
DGCNN	0.676	0.804	0.906	0.545	0.268	0.898	0.488	0.415	0.773	0.810	0.641
DGCNN-voxel	0.577	0.788	0.901	0.733	0.250	0.913	0.425	0.430	0.792	0.813	0.645
GAFFNet	0.587	0.818	0.916	0.775	0.348	**0.941**	**0.603**	0.447	**0.821**	**0.841**	0.695

**Table 6:** OA and macro avg F1 scores of all ablated networks.

Ablation Studies	OA	Macro Avg F1
(1) max pooling	0.834	0.693
(2) sum pooling	0.833	0.689
(3) mean pooling	0.827	0.680
(4) GAFFNet-RS	0.812	0.633
(5) one NFFU	0.815	0.662
(6) three NFFUs	0.832	0.685
(7) no height above DTM	0.835	0.699
(8) no statistical features	0.836	0.672
GAFFNet	0.841	0.695

## Data Availability

Not applicable.
